# On the Combined Action of Morphia and Chloroform

**Published:** 1870-10

**Authors:** Claude Bernard


### On the Combined Action of Morphia and Chloroform.

BY PROF. CLAUDE BERNARD.

In his very interesting lectures before the College of France, Professor Claude Bernard points out some remarkable and valuable effects which may be obtained by combining the actions of morphia and chloroform (*Revue des Cours Scientifiques;* and *Bulletin General Therapeutique*, Sept. 30. 1869, p. 241). Some years ago, Dr. Bernard had occasion to administer a dose of morphia to a dog recovering from the effects of chloroform, and he was surprised to observe that the morphia reproduced the anæsthetic effect of the previous dose of chloroform. More recently this experiment was modified, so that a dog narcotized by morphia was completely and quickly anæsthetized by a quantity of chloroform very much smaller than would have been necessary to produce this effect in a dog in normal condition. It was further found, that when the anæsthesia had nearly disappeared, a second dose of morphia almost immediately reproduced it. It is wèll known that anæsthesia cannot be induced by morphia. This alkaloid exalts the sensory excitability, and induces torpor and narcotism; it never destroys sensibility. Chloroform, however, rapidly suspends the sensory excitability. It is, therefore, somewhat remarkable that the anæsthetic action of chloroform should be increased and prolonged by morphia. Dr. Bernard believes that this can be explained only by supposing that the action of the one substance is superimposed on that of the other. When an animal is recovering from chloroform-anæsthesia, the quantity present in the blood is insufficient to completely suspend sensibility, although it is sufficient to great diminish it; but as morphia blunts the nervous sensibility, it aids the action of the chloroform, and thus a quantity of the latter, in itself insufficient to produce complete anæsthesia, becomes sufficient to do so when assisted by morphia. Bernard points out that this combination admits of several valuable applications, and that, indeed, it promises to be the best method of inducing anæsthesia. By administering a dose of morphia before commencing the inhalation of chloroform, anæsthesia is induced without any initiatory stage of excitement and without incurring the risk of those accidents which are occasionally caused by a large dose of chloroform.—*Jour. Anat. and Phys.*, Nov. 1869.—(*Half-Yearly Abstract.*)

